# Evaluation of Endovascular Treatment for Acute Basilar Occlusion in a State-Wide Prospective Stroke Registry

**DOI:** 10.3389/fneur.2021.678505

**Published:** 2021-06-11

**Authors:** Katharina Gruber, Björn Misselwitz, Helmuth Steinmetz, Waltraud Pfeilschifter, Ferdinand O. Bohmann

**Affiliations:** ^1^Department of Neurology, University Hospital/ Goethe University Frankfurt, Frankfurt, Germany; ^2^Geschäftsstelle Qualitätssicherung Hessen, Frankfurt, Germany

**Keywords:** thrombectomy, best medical treatment, posterior circulation, thrombolysis, endovascular treatment, basilar artery occlusion (BAO)

## Abstract

**Context:** Despite overwhelming evidence for endovascular therapy in anterior circulation ischemic stroke due to large-vessel occlusion, data regarding the treatment of acute basilar artery occlusion (BAO) are still equivocal. The BASICS trial failed to show an advantage of endovascular therapy (EVT) over best medical treatment (BMT). In contrast, data from the recently published BASILAR registry showed a better outcome in patients receiving EVT.

**Objective:** The aim of the study was to investigate the safety and efficacy of EVT plus BMT vs. BMT alone in acute BAO.

**Methods:** We analyzed the clinical course and short-term outcomes of patients with radiologically confirmed BAO dichotomized by BMT plus EVT or BMT only as documented in a state-wide prospective registry of consecutive patients hospitalized due to acute stroke. The primary endpoint was a favorable functional outcome (mRS 0–3) at hospital discharge assessed as common odds ratio using binary logistic regression. Secondary subgroup analyses and propensity score matching were added. Safety outcomes included mortality, the rate of intracerebral hemorrhages, and complications during hospitalization.

**Results:** We included 403 patients with acute BAO (2017–2019). A total of 270 patients (67%) were treated with BMT plus EVT and 133 patients (33%) were treated with BMT only. A favorable outcome (mRS 0–3) was observed in 33.8% of the BMT and 26.7% of the BMT plus EVT group [OR.770, CI (0.50–1.2)]. Subgroup analyses for patients with a NIHSS score > 10 at admission to the hospital revealed a benefit from EVT [OR 3.05, CI (1.03–9.01)].

**Conclusions:** In this prospective, quasi population-based registry of patients hospitalized with acute BAO, BMT plus EVT was not superior to BMT alone. Nevertheless, our results suggest that severely affected BAO patients are more likely to benefit from EVT.

## Introduction

Arterial occlusions of the posterior intracranial circulation account for about 20% of all ischemic strokes, and of these, an estimated 15% are due to basilar artery occlusion (BAO) (1). The spontaneous course of BAO is associated with high mortality and morbidity mostly leading to poor patient outcome. Evidence for effective therapies is scarce (2). Whereas the benefit of endovascular therapy (EVT) in anterior circulation ischemic strokes due to large-vessel occlusion has been proven by several randomized trials, (3–7) the efficacy of EVT over standard medical care has not yet been unequivocally shown in BAO patients. Recently, two randomized-controlled trials failed to prove additional benefit of EVT in patients with acute BAO (8, 9). By contrast, a consecutive registry of patients with angiographically proven BAO showed that patients receiving EVT tend to benefit in terms of functional recovery after 90 days, whereas the differences in thrombolytic treatment and process times should be noted (10).

We aimed to investigate the safety and efficacy of EVT plus best medical treatment (BMT) vs. BMT alone in acute BAO using consecutive, quasi population-based, real-life data from our mandatory state-wide quality assurance registry.

## Materials and Methods

We retrieved data from a mandatory prospective stroke inpatient quality assurance registry covering the entire federal state of Hessen in Germany (6,285,000 inhabitants). The register represents the complete hospital landscape of the state of Hesse, i.e., a total of 119 hospitals. Data entry is compulsory by a federal contract and the registry achieves a nearly 100% completion, verified by administrative hospital data. Due to the anonymized data collection in the context of quality assurance measures, individual consent and ethical votes were not required.

We included patients fulfilling the following criteria: (1) discharge diagnosis of ischemic stroke (ICD-10: I63), (2) age 18 years or older, and (3) BAO confirmed by computed tomographic angiography, magnetic resonance angiography, or digital subtraction angiography. Estimated BAO time was recorded from symptom onset to the arrival time at the hospital. In case the symptom onset was not known, the time of last seen well was assumed instead. If no last seen well could be determined, the duration of symptom onset to admission was noted as unknown.

We collected information on baseline characteristics, stroke risk factors, stroke severity, and neurological deficits at presentation, pre- and post-treatment angiographic findings, process times, type of treatment, and functional outcomes at discharge for three subsequent years (2017–2019).

We distinguished between patients who received BMT (e.g., intravenous thrombolysis, coagulation management, and blood pressure management) and patients who additionally underwent endovascular thrombectomy (BMT + EVT). The primary clinical efficacy outcome was a favorable outcome defined as modified Rankin scale (mRS) from 0 to 3 points at discharge from hospital. The mRS is a seven-level scale [range, 0 (no symptoms) to 6 (death)] for the assessment of neurologic functional disability (11). Common odds ratio for a shift in scores on the mRS was calculated by ordinal logistic regression.

The main secondary clinical efficacy outcome was good or excellent functional outcome at discharge from hospital (mRS 0–2/0–1). As safety outcomes mortality and complications during hospital stay were recorded.

### Statistical Analysis

We compared baseline characteristics, treatment metrics, outcomes, and severe adverse events between the BMT-alone and BMT plus EVT group. Data are presented as means [standard deviation (SD)] if normal distributed or medians [interquartile ranges (IQRs)] or numbers with percentages, unless otherwise indicated.

Univariate analysis was performed using the Mann–Whitney *U* test, χ^2^ test, as appropriate. The primary outcome variable was the adjusted common odds ratio for a favorable outcome on the mRS score (0–3 points); this ratio was estimated with binary logistic regression. The adjusted common odds ratios are reported with 95% CIs to indicate statistical precision.

Adjusted estimates of outcome (common odds ratio, odds ratio, and β) were calculated by taking the following variables into account: age, baseline NIHSS, sex, and intravenous thrombolysis (IVT).

For propensity score matching analysis, we performed a 1:1 matching based on the nearest-neighbor matching algorithm with a caliper width of 0.2 of the propensity score with age, care situation prior admission (independent at home, care at home, and care in institution), mRS at admission, baseline NIHSS, and medical history, such as diabetes mellitus, arterial hypertension, and prior stroke. In line with randomized trials of acute stroke therapies, only patients with an independent lifestyle and time from onset to admission <24 h were included for propensity score matching to exclude comorbidities as potential influence factors.

The significance level was set to *P* < 0.05, and all tests of hypotheses were two-sided. Data were analyzed with SPSS 26 (IBM; Armonk, BY, USA) and GraphPad 9 (GraphPad Software, USA).

## Results

### Baseline Characteristics

In total, 403 patients with acute BAO were included. A total of 270 patients (67%) were treated with BMT plus EVT and 133 patients (33%) were treated with BMT alone (Table 1). Patients from the BMT group were slightly older (73.4 ± 13.1 years, *p* = 0.06) than those in the BMT plus EVT group (71.1 ± 12.9 years, *p* = 0.06) and fewer patients in this group were functionally independent before the stroke (75.2 vs. 84.1%, *p* = 0.008). The burden of vascular risk factors (diabetes and hypertension) and previous stroke was numerically but non-significantly higher in the BMT-only group. The median NIHSS at admission was significantly higher in the BMT plus EVT group (14 points vs. 8 points, *p* < 0.001) and more patients showed dysarthria (*p* = 0.05) and dysphagia (*p* = 0.002). Overall, 33.1% of patients in the BMT and 16.6% in the BMT plus EVT group received IVT with a mean door-to-needle time of 21.0 min (±24.1, BMT) and 23.9 min (±21.5, *p* = 0.27) and a mean door-to-groin time of 69.9 min (±87.3, BMT plus EVT).

**Table 1:** Baseline characteristics upon hospital admission and process measures.

	**Overall**			**Propensity Score Matched**		
	**BMT *n* = 133**	**BMT + EVT *n* = 270**	***p***	**BMT *n* = 73**	**BMT + EVT *n* = 73**	***p***
Age (years, mean, SD)	73.4 ± 13.1	71.1 ± 12.9	0.06	69.86 (±13.2)	68.3 (±13.3)	0.485
Female	68 (51.1)	114 (42.2)	0.347	33 (45.2)	31 (42.5)	0.868
NIHSS (median, IQR)	8 (3–20)	14 (7–22)	<0.001	6 (3–17)	8 (4–18)	0.255
NIHSS <10	72 (54.1)	94 (34.8)	<0.001	45 (61.6)	37 (50.7)	0.243
NIHSS ≥ 10	61 (45.9)	176 (65.2)	<0.001	28. (38.4)	36 (49.3)	0.243
Need for care prior stroke			0.008			
- independent- care at home- institutional care	100 (75.2) 11 (8.3) 22 (16.5)	227 (84.1) 25 (9.3) 18 (6.7)		100 (100) —-	100 (100) –	–
Admission to hospital			0.218			0.776
- self-initiated- *via* family physician- *via* emergency service- secondary transfer	3 (2.3) 4 (3.0) 95 (71.4) 31 (23.3)	2 (0.7) 5 (1.9) 180 (66.7) 83 (30.7)		3 (4.1) 1 (1.4) 50 (68.5) 19 (26.0)	0 (0) 1 (1.4) 51 (69.9) 21 (28.8)	
OAT prior admission			0.364			0.748
- no OAT- Vitamin K Antagonist- NOAC	118 (88.7) 5 (3.8) 10 (7.5)	220 (81.5) 18 (6.7) 32 (11.9)		65 (89.0) 4 (5.5) 4 (5.5)	62 (84.9) 6 (8.2) 5 (6.8)	
**Risk factors**						
Previous stroke	33 (24.8)	60 (22.2)	0.348	17 (23.3)	14 (19.2)	0.851
Hypertension	106 (79.7)	202 (74.8)	0.241	58 (79.5)	60 (82.2)	0.834
Diabetes	38 (28.6)	65 (24.1)	0.441	19 (26.0)	21 (28.8)	0.853
**Stroke symptoms**						
Aphasia	31 (23.3)	71 (26.3)	0.221	13 (17.8)	13 (17.8)	1.0
Dysarthria	72 (54.1)	149 (55.2)	0.050	37 (50.7)	40 (54.8)	0.740
Dysphagia	51 (38.3)	128 (47.4)	0.002	23 (31.5)	34 (46.6)	0.089
Paresis	77 (57.9)	154 (57.0)	0.336	39 (53.4)	28 (38.4)	0.331
**Onset to admission**						
0–6 h 6–24 h >24 h unknown	66 (49.6) 30 (22.6) 16 (12.0) 21 (15.8)	177 (65.6) 46 (17.0) 10 (3.7) 37 (13.7)	0.472 0.223 0.002 0.549	44 (60.3) 29 (39.7) – –	43 (58.9) 30 (41.1) –	0.99 0.99 – –
**Treatment**						
Thrombolysis	44 (33.1)	45 (16.6)	0.001	30 (41.1)	37 (50.7)	0.319
Endovascular treatment– TICI IIB/III	–	270 (100) 200 (74.1)	–	—-	100 (100) 55 (75.3)	–
Door-to-needle (mean, SD)Door-to-imaging (mean, SD)Door-to-groin (mean, SD)	21.0 ± 24.1 25.7 ± 38.2 –	23.9 ± 21.5 20.1 ± 26.8 69.9 ± 87.3	0.274 0.842 –	18.0 ± 17.3 19.4 ± 27.7 –	23.9 ± 24.9 25.2 ± 33.7 76.2 ± 90.0	0.360 0.371 –
Length of hospitalization (day, mean, SD)	10.8 ± 12.5	12.1 ± 13.2	0.337	12.9 ± 15.1	12.3 ± 8.8	0.773

*BMT, best medical treatment; EVT, endovascular thrombectomy; IQR, interquartile range; NIHSS, National Institutes of Health Stroke Scale; mRS, modified rankin scale; OAT, oral anticoagulation treatment; NOAC, novel oral anticoagulant; TICI, thrombolysis in cerebral infarction scale, unless otherwise declared n (%)*.

### Primary and Secondary Efficacy Outcomes

**Figure 1:**
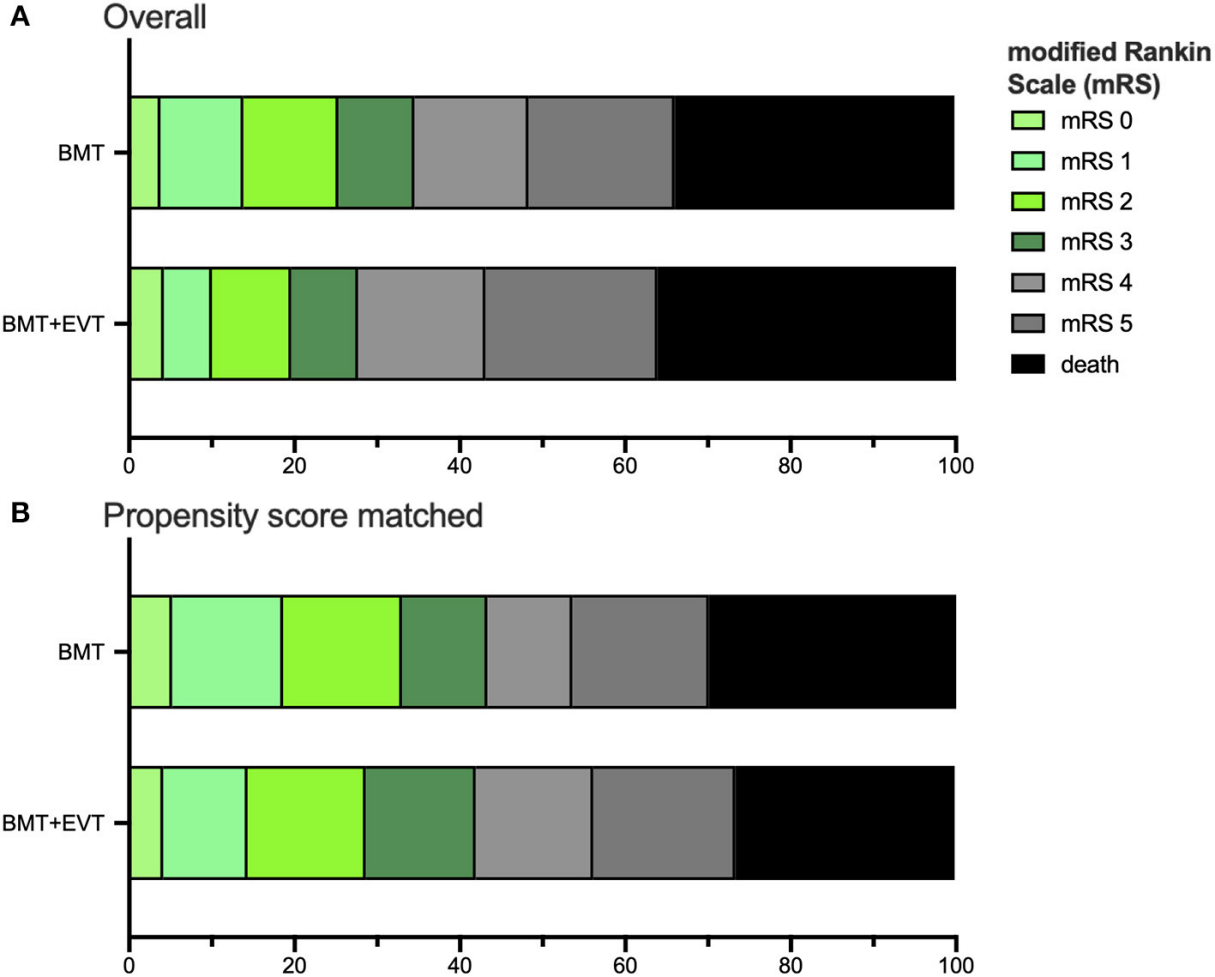
Distribution of the modified Rankin scale (mRS) in all patients **(A)** and the Propensity Score matched data set **(B)** for age, mRS at admission, baseline NIHSS, and medical history, such as diabetes mellitus, arterial hypertension, and prior stroke. Comparing BMT vs. BMT plus EVT group, the mRS shift was not significant, neither for the all patients [OR 0.81 (0.56–1.19)] nor for Propensity Score matched patients [OR 0.98 (0.60–1.61)]. The ratio was estimated with ordinal logistic regression.

Of the patients in the BMT group and the BMT plus EVT group, 33.8 and 26.7%, respectively, reached favorable outcome (mRS 0–3) [OR 0.770, CI (0.50–1.20)]. The median short-term mRS at discharge after a median length of stay of 9 days (IQR 4–16 days) was 5 and did not differ between BMT and BMT plus EVT (*p* = 0.28, Table 2). Common odds ratio for a shift in scores on the mRS was not significant comparing BMT vs. BMT plus EVT [OR 0.81 (0.56–1.19), Figure 1A].

**Table 2:** Primary and secondary efficacy outcomes and safety outcomes.

	**Overall**			**Propensity Score Matched**		
	**BMT**	**BMT + EVT**		**BMT**	**BMT + EVT**	
	***n* = 133**	***n* = 270**	***p***	***n* = 73**	***n* = 73**	***p***
**Primary Outcome (*****n*****, %)**						
mRS 0–3	45 (33.8)	87 (26.7)	0.248	29 (41.4)	31 (43.1)	0.844
**Secondary Outcome (*****n*****, %)**						
mRS 0–2	33 (24.8)	51 (18.9)	0.323	23 (32.9)	19 (26.4)	0.398
mRS 0–1	18 (13.5)	26 (9.6)	0.506	14 (20.0)	9 (12.5)	0.225
mRS (median, IQR)	5 (2–6)	5 (3–6)	0.284	4 (2–6)	4 (2–5)	0.962
**mRS (median, IQR)**						
0–6 h onset to admission6–24 h onset to admission>24 h onset to admissionunknown onset	5 (3–6) 4 (2–5) 3 (2–6) 6 (4–6)	5 (3–6) 5 (4–6) 4.5 (2–6) 5 (4–6)	0.952 0.496 0.517 0.479	4 (2–6) 4 (2–5) –	4 (2–5) 4 (3–6) –	0.4670.330 –
**mRS (median, IQR)**						
<10 NIHSS on admission≥10 NIHSS on admission	3 (1.75–5) 6 (5–6)	3 (2–5) 5 (4–6)	0.3510.037	3 (1–5) 5 (5–6)	3 (1–5) 5 (4–6)	0.9400.207
**mRS (median, IQR)**						
<70 years≥70 years	4 (2–6) 5 (4–6)	5 (2–6) 5 (4–6)	0.5020.358	4 (2–5) 4.5 (2–6)	4 (2–5) 4 (3–6)	0.6770.685
**Safety outcome (*****n*****, %)**						
Mortality	44 (33.8)	94 (36.2)	0.730	18 (24.7)	14 (19.1)	0.549
Surgical decompression	3 (2.3)	11 (4.1)	0.336	3 (4.1)	2 (2.7)	0.999
Pneumonia	109 (82.0)	73 (27.0)	0.047	15 (20.5)	17 (23.3)	0.842
Brain edema	7 (5.3)	24 (8.9)	0.199	5 (6.8)	5 (6.8)	1
Intracerebral bleeding	1 (0.8)	4 (1.5)	0.534	0 (0)	2 (2.7)	0.497
Cerebral arterial embolism	4 (3.0)	13 (4.8)	0.396	2 (2.7)	4 (5.5)	0.681

*BMT, best medical treatment; EVT, endovascular thrombectomy; IQR, interquartile range; NIHSS, National Institutes of Health Stroke Scale; mRS, modified rankin scale*.

**Figure 2:**
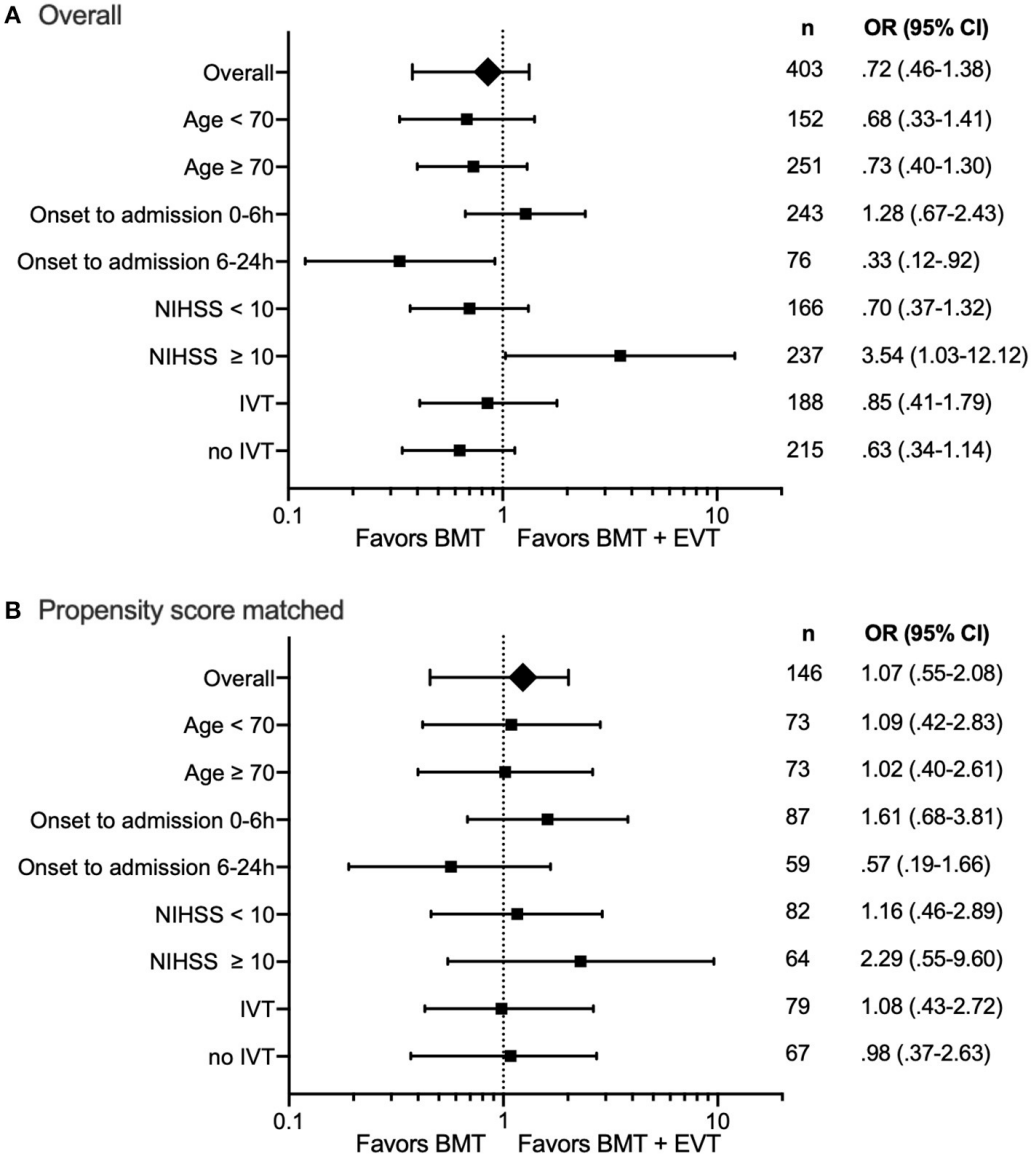
Forest plot of additional endovascular therapy (EVT) on favorable outcome (mRS 0–3) at discharge from hospital. **(A)** includes all patients (*n* = 403). **(B)** Propensity Score matched data set (*n* = 146) for age, care situation prior to admission (independent at home, care at home, and care in institution), mRS at admission, baseline NIHSS, and medical history, such as diabetes mellitus, arterial hypertension, prior stroke, and time from onset to admission <24 h. Number of patients and odds ratio (OR) with 95% CI are presented.

Good functional outcome, defined as mRS 0–2 at discharge, was reached by 24.8% of the BMT and 18.9% of the BMT plus EVT group (*p* = 0.32). In patients with a severe stroke (NIHSS ≥ 10), median mRS at discharge was 6 in patients of the BMT and 5 in patients of the BMT plus EVT group (*p* = 0.04). Results of the subgroup analysis are presented in Table 2. Regarding the best treatment option for patients with a severe stroke, there was a significant signal for a benefit from additional EVT [OR 3.05, CI (1.03–9.01), Figure 2]. A further subgroup analysis showed that patients with an onset to hospital admission time from 6 to 24 h achieved a significantly better outcome by BMT alone compared to BMT plus EVT [OR 0.33 (0.12–0.92)].

### Propensity Score Matched

After propensity score matching, the baseline characteristics of the BMT and BMT plus EVT group were well-balanced (Table 1). There was no significant difference in either the primary or secondary endpoints (Table 2). Common odds ratio for a shift in scores on the mRS was not significant comparing BMT vs. BMT plus EVT [OR 0.98 (0.60–1.61), Figure 1B]. Favorable outcome (mRS 0–3) was reached in 42% within the BMT and in 41% within the BMT plus EVT group (*p* = 0.78). Additional adjustments for age or stroke severity also did not uncover significant discrepancies between the two groups.

### Safety Outcomes

Mortality was high in both groups (33.8 vs. 36.2%, *p* = 0.73). Patients of the BMT plus EVT group showed a statistically non-significant higher rate of intracerebral bleeding complications (1.5 vs. 0.8%, *p* = 0.53) and were more frequently treated in intensive care units (82.6 vs. 42.9%, *p* < 0.001). Besides, we observed a lower rate of pneumonia in this group (27.0 vs. 82%, *p* = 0.05).

## Discussion

The present study provides consecutive and non-selective prospective real-world data on the management and treatment results of patients with acute BAO. At the time of hospital discharge, there was no significant difference in terms of favorable outcome (mRS 0–3) between patients treated with BMT alone and patients treated with BMT and EVT. However, our data suggest that patients with severe stroke might benefit from additional EVT.

These results are in line with recently presented results of the randomized-controlled BASICS trial conducted in the Netherlands, Switzerland, Germany, Italy, Norway, and Brazil, which randomized 300 patients from 2011 to 2019 with a BAO (<6 h since onset) to best medical management plus EVT or best medical management alone (8). The primary endpoint of favorable outcome defined as mRS ≤ 3 after 90 days did not differ significantly between groups (oral presentation ESOC 2020, Vienna, Austria, https://eso-wso-conference.org/eso-wso-may-webinar/). The authors discussed a higher-than-expected rate of favorable outcome in the BMT-only group (37.7% reached mRS 0–3 after 90 days) as one of the factors for the neutral result of the trial. Furthermore, regarding the prespecified subgroup analysis, the authors stated that especially in severely affected patients (NIHSS > 10), EVT might be more effective, whereas in NIHSS, <10 BMT seems to be superior.

The randomized-controlled BEST trial conducted in China was terminated early after enrollment of 131 patients due to slow recruitment and a high crossover rate into the EVT arm (9). The primary intention-to-treat analysis was neutral despite numerically more patients reaching mRS ≤ 3 at 90 days. There was no effect of additional EVT on mortality in the primary analysis. Secondary “per protocol” and “as treated” analyzing crossover patients in the EVT pointed toward a superior efficacy of EVT. Of note, there was a numerically higher rate of hemorrhagic complications in the arm with additional EVT (9).

BASILAR, a large Chinese registry that consecutively enrolled 829 adult patients with angiographically proven acute BAO with symptom onset <24 h, showed a clear treatment preference toward additional endovascular therapy that was delivered to 78% of patients (10). A non-selected analysis as well as an analysis after propensity score matching for age, systolic blood pressure, baseline pc-ASPECTS, baseline NIHSS, TOAST classification, occlusion site, and medical history found significantly better outcomes in a mRS shift analysis and significantly more patients with mRS ≤ 3 at 90 days as well as a significantly lower mortality in patients receiving additional EVT. With a median NIHSS of 27 points, patients in the BASILAR registry were more severely affected than in the BASICS study (median 21 and 22, respectively) and significantly more severely affected than in our registry. Together with the indications of a higher additional efficacy of EVT in more severely affected patients with BAO from the BASICS trial, we hypothesize that the differences between this and the present registry data might be at least partially attributable to the higher rate of severe strokes in the BASILAR registry. This is supported by a relevantly lower mortality rate in our registry that did not differ significantly between both treatment groups. Given the quasi-population based nature of our registry data from a mandatory quality assurance database, we think that our data are representative for a central European stroke patient population.

When considering both registries, it should be noted that our patients were included for the most part up to 24 h after symptom onset, and, in addition, in a small part, no symptom onset was known at all, differing from the randomized-controlled trials BASICS and BEST with intervals up to 6 and 8 h after onset, respectively. The currently ongoing randomized BAOCHE trial conducted at multiple sites in China aims to shed more light on the efficacy of additional EVT in the late time window.

The three sources of evidence discussed above (BASICS, BEST, and BASILAR) and our data do not show an excess risk of additional EVT in patients with acute BAO of all degrees of severity. A secondary analysis from the BASICS trial as well as our analysis dichotomizing the population by an NIHSS score of 10 suggest a superior efficacy of additional EVT in patients with acute BAO and severe stroke. Looking at the subgroup analysis of our study, for patients with symptom onset to admission 6–24 h, BMT appears superior. Nevertheless, we believe that the data of our study are not sufficient to argue in principle against EVT beyond 6 h. This is also supported by the fact that the effect was no longer detectable after propensity score matching. In contrast to our study, the BASILAR study could show an efficacy of EVT for this subgroup (10).

A limitation of our study is the fact that our quality assurance database only captures the inpatient stay as we do not have access to the functional status beyond the mRS at discharge. On the other hand, our data reveal population-based information on acute treatment of BAO with little selection bias. In addition, an observation period of 3 months may also be too short to adequately reflect recovery in the most severely affected patients.

Recent data on the use of tenecteplase for BAO and consecutive EVT have demonstrated an increased likelihood of reperfusion (12). However, based on our data, we cannot comment on this because the type of thrombolytic agent used is not recorded.

In summary, our study showed no significant difference between BMT plus EVT vs. BMT alone on short-term functional outcome. Taking into account the recently published BASICS and BEST trials and the data from the BASILAR registry, it can be assumed that additional EVT is safe and that severely affected patients seem to benefit from additional EVT. Further clinical studies are necessary to better define the patient population with a high likelihood of clinical benefit from additional EVT for acute BAO.

## Data Availability Statement

The datasets generated for this article are not readily available because due to legal restrictions, the data cannot be made available. Requests to access the datasets should be directed to Dr. Ferdinand Bohmann, ferdinand.bohmann@kgu.de.

## Ethics Statement

Ethical review and approval was not required for the study on human participants in accordance with the local legislation and institutional requirements. Written informed consent for participation was not required for this study in accordance with the national legislation and the institutional requirements.

## Author Contributions

FB and WP conceived the study. FB and KG managed the data and performed the statistical analysis. BM runs the state-wide prospective quality assurance database. All authors contributed to manuscript revision, and read and approved the submitted version.

## Conflict of Interest

The authors declare that the research was conducted in the absence of any commercial or financial relationships that could be construed as a potential conflict of interest.
